# Opinion: Endoscopic Urethrotomy

**DOI:** 10.1590/S1677-5538.IBJU.2015.04.03

**Published:** 2015

**Authors:** André G. Cavalcanti, Gustavo Fiedler

**Affiliations:** 1 Universidade Federal do Rio de Janeiro, RJ, Brasil; 2 Hospital Federal Cardoso Fontes, Rio de Janeiro, RJ, Brasil; 3 Hospital dos Servidores Federais, Rio de Janeiro, RJ, Brasil

**Keywords:** Urethral Stricture, Anastomosis, Surgical, Urethra, Endoscopy

Urethral stricture is one of the most difficult urological problems to heal adequately and many surgical and no surgical techniques have been described to its management. Reports has been documented in ancient literature of the Hindus, Egyptians and Greeks (1). In 1957, Ravasini described the internal urethrotomy under direct vision and used electrocautery to incise the stricture. In 1971, Sachse introduced the sharp-bladed cold-knife urethrotome under direct vision, reporting 80% success rate with this procedure in 1974 (1, 2).

Since this description by Sachse, the direct vision internal urethrotomy (DVIU) became one of the most popular treatments on the management of urethral strictures. The male anterior urethra stricture is characterized by a fibrotic process which achieves varying degrees of peri-urethral spongiosum tissue fibrosis (spongiofibrosis) associated with a decrease in urethral caliber and consequently in the urine flow. Endoscopic treatment is based on the deep incision of the fibrotic area and subsequently local reepithelization process to the maintenance of the urethral patency.

Data from the National Health Service in the UK during 2006 showed that the DVIU or urethral dilatation was used in 93% of cases and urethroplasty in 7% of cases (1). In a nationwide survey in the Netherlands, the DVIU was practiced by 97% of urologists. Urethroplasty was performed at least once yearly by 23%, with only 6% performing more than five urethroplasties annually (3). A similar date was observed from an US survey. Most urologists (63%) treat between 6-20 urethral strictures per year. The most common minimally invasive procedures used for anterior urethral strictures managing were urethral dilation (92.8%), cold-knife optical internal urethrotomy (85.6%), endourethral stents (23.4%), laser urethrotomy (19%), and periurethral steroid injection after urethrotomy (7.9%) (4).

During the 80's and 90's decades, many authors have published very interesting rates of success for the endoscopic management of anterior urethral strictures and helped to popularize this method in this period. Even in cases of complete obstruction of the urethral, with intense spongiofibrosis, the use of the DVIU was postulated by some authors. Publications demonstrating a success rate up to 77% on 369 procedures performed in 225 patients (5), or 85% of success, requiring repeat procedures in 33% of cases (6) are examples of those results. Reviewing the literature, Passadoro and Emiliozzi observed a success rate for the DVIU from 56-96% in 23 articles (7). The authors also observed the lack of standardization regarding the criteria for success evaluation - only subjective for some authors and according to the uroflometry for others. And when the uroflometry was used, different maximun flow rate cut offs were defined (7)

Another factor that contributed to increase the use of the DVIU among urologists was the possibility to be performed in a completely outpatient basis, especially under local or loco-regional anesthesia. For this technical facilities, many urologists belive that performing multiple endoscopic procedures would have benefits over more invasive sur-gical procedures such an urethroplasty.

Despite the good results published initially, studies presented in the late 90s and early in this century, demonstrated that the success rates for the DVIU were more limited, specially during a long period of observation. The overall success rate of internal urethrotomy for anterior urethral strictures is 32-40 % with long-term (>24 months) follow-up (7–10) risk factors for failure include urethral stricture of the penis (vs. bulbar), long lesions (>1 cm or >2 cm ranging according the study) and traumatic etiology. The best results were observed for the first endoscopic procedure and the success rate comes to 0 % on multiple interventions (3 or >) (7, 10).

More recently, Santucci et al. presented lower success rates for the DVIU. In this study, the success rates were no higher than 9% for first or subsequent urethrotomy during the observation period (11).

As a treatment for male urethral stricture, internal urethrotomy has the advantages of ease, simplicity, speed and short convalescence. Despite that, some rate of complications is associated to the procedure– erectile dysfunction, penile curvature, bleeding, urinary tract infections and priapism (5–11).

Various modifications of the classic single cold-knife incision in the 12 o'clock position have been proposed, including multiple radial incisions. Another point, is the post operative period with the urethral catheter. In both, number or position of the incisions and time of catheterization, the publications are usually retrospective case-series and there are no good prospective, randomized studies to prove their claims of greater efficacy. Our personal preference is to use multiple radial incisions (between 3–5) to improve the reepithelization area and leave a silicon catheter for 3 days only. In a survey with American Urologic Association members, the majority of the urologists leave the catheter around one week (4).

In recent decades several authors have published more significant changes in technique, mainly related to new forms of energy and the use of anti-fibrotic agents.

Lasers have been used for the treatment of urethral strictures since 1977. The types used for urethrotomy include carbon dioxide, Nd: YAG, the KTP, the Argon, the Ho: YAG and excimer lasers (1), but most of the time described in low quality studies. Comparing the DVIU using Holmium Laser with classic urethrotomy with a cold knife, Dutkiewicz and Wroblewski didin't observe differences in terms of safety and efficacy (12). In another randomized study, Jain et al observed that both modalities are effective in providing immediate relief of the symptoms to patients with single and short segment urethral strictures (<2 cm long). A more sustained response was attained with cold knife urethrotomy (1).

The use of various anti-fibrotic substances during the endoscopic procedure have also been describe by many authors - corticosteroids, botulinum toxin, hyaluronic acid, captopril gel and mitomycin C among others. A systematic review showed that the use of local corticosteroid may prolong the time to recurrence of stenosis after a DVIU but not seem to affect the final rate of recurrence (13). Kim et al observed no advantage in the use of intra-urethral instillations of hyaluronic acid on the results of classic DVIU in the literature (14). A phase II study showed advantages in the use of gel captopril compared to the placebo group (15). A prospective randomized study with 40 patients, specifically focused on anterior urethral strictures, described advantage with the use of submucosal injection of mitomycin C during the DVIU (16). More recently, the use of injection with a combination of substances (triamcinolone, mitomycin C and hyaluronic acid) has also been proposed, showing safety and effectively in a short follow-up (17). Although a number of studies demonstrating the safety and viability of adjuvant local therapy for the DVIU there is a lack of robust data with randomized trials and significant follow-up.

In our practice we found that ultrasound can be used as a complementary method to the retrograde and voiding urethrogram in selecting patients for an internal urethrotomy. This method can provide more accuracy in defining the length and location of the stricture segment and can add information about the degree of spongiofibrosis and the urethral caliber. The proper selection and characterization of patients seems to be essential to achieve better results with the internal urethrotomy.

A common practice among urologist, is the use of sequential dilatation protocols after the DVIU, which can reduce the chance of failure, as described in some papers (10). From the point of view of most of urethral reconstructive surgeons, to perform dilation, that is already considered a failure of the initial approach, can add risks to patients and decrease the quality of life with repeated procedures. Repeated endoscopic procedures will not only delay cure, but may also worsen stricture characteristics by increasing the length of the stricture and causing more local fibrosis (10, 18).

Wright et al. indicated that the most cost-effective management algorithm for a bulbar urethral stricture of <2 cm is a single internal urethrotomy followed by urethroplasty if the urethrotomy fails (18). Rourke and Jordan observed that the treatment for short segment bulbous urethral strictures with primary reconstruction is less costly than treatment with DVIU (19).

In the group of patients selected to have the best results for the internal urethrotomy (first procedure, short, bulbar and no traumatic strictures) the results observed to the urethroplasty still seems to be more effectives. Failures for urethroplasty, in this group, will be an uncommon event. Excision with primary urethral anastomosis has a high success rate with durable long-term results in most patients and the complications are rare with short duration and self-limited. (9, 10, 11).

The DVIU can also be an option in the initial management of localized failures after urethroplasty, including procedures with tissue substitution (grafts and flaps). In a retrospective study, Rosenbaum et al observed that about half of patients with re-strictures <1 cm after uretroplasties with buccal mucosa grafts (BMG) can be treated with a internal urethrotomy without new failures in a mean follow-up of 11.7 months (20).

In our practice we consider to offer internal urethrotomy for selected cases as first surgical alternative - bulbar, short (<1 cm) and non-traumatic (with little or minimal spongiofibrosis). All patients should be informed about the low success rates in the long term follow-up with this procedure and the possibility to be submitted to procedures with more definitive results as anastomotic urethroplasty or substitution urethroplasty. We do not offer a second urethrotomy as an option after an initial failure. The internal urethrotomy may also be used as a optional technique in some patients with post urethroplasty failures and even in patients with high surgical risk for a more aggressive procedures.

Urethral dilatation and DVIU remain widely used in urethral stricture management but high-level comparative evidence of benefit and harms against urethroplasty in the short and long-term is still lack.
